# Assessment of Preoperative Liver Function in Patients with Hepatocellular Carcinoma – The Albumin-Indocyanine Green Evaluation (ALICE) Grade

**DOI:** 10.1371/journal.pone.0159530

**Published:** 2016-07-19

**Authors:** Takashi Kokudo, Kiyoshi Hasegawa, Katsumi Amikura, Emilie Uldry, Chikara Shirata, Takamune Yamaguchi, Junichi Arita, Junichi Kaneko, Nobuhisa Akamatsu, Yoshihiro Sakamoto, Amane Takahashi, Hirohiko Sakamoto, Masatoshi Makuuchi, Yutaka Matsuyama, Nicolas Demartines, Massimo Malagó, Norihiro Kokudo, Nermin Halkic

**Affiliations:** 1 Hepato-Biliary-Pancreatic Surgery Division and Artificial Organ and Transplantation Division, Department of Surgery, Graduate School of Medicine, The University of Tokyo, Tokyo, Japan; 2 Division of Gastroenterological Surgery, Saitama Cancer Center, Saitama, Japan; 3 Department of Visceral Surgery, University Hospital Centre Hospitalier Universitaire Vaudois, Lausanne, Switzerland; 4 Division of Hepato-Billiary-Pancreatic Surgery, Japanese Red Cross Medical Center, Tokyo, Japan; 5 Department of Biostatistics, School of Public Health, The University of Tokyo, Tokyo, Japan; 6 Department of Surgery-UCL Division of Surgical and Interventional Sciences, University College London, London, United Kingdom; ISMETT-UPMC Italy/ University of Catania, ITALY

## Abstract

**Background:**

Most patients with hepatocellular carcinoma (HCC) have underlying liver disease, therefore, precise preoperative evaluation of the patient’s liver function is essential for surgical decision making.

**Methods:**

We developed a grading system incorporating only two variables, namely, the serum albumin level and the indocyanine green retention rate at 15 minutes (ICG R15), to assess the preoperative liver function, based on the overall survival of 1868 patients with HCC who underwent liver resection. We then tested the model in a European cohort (n = 70) and analyzed the predictive power for the postoperative short-term outcome.

**Results:**

The Albumin-Indocyanine Green Evaluation (ALICE) grading system was developed in a randomly assigned training cohort: linear predictor = 0.663 × log_10_ICG R15 (%)−0.0718 × albumin (g/L) (cut-off value: -2.20 and -1.39). This new grading system showed a predictive power for the overall survival similar to the Child-Pugh grading system in the validation cohort. Determination of the ALICE grade in Child-Pugh A patients allowed further stratification of the postoperative prognosis. This result was reproducible in the European cohort. Determination of the ALICE grade allowed better prediction of the risk of postoperative liver failure and mortality (ascites: grade 1, 2.1%; grade 2, 6.5%; grade 3, 16.0%; mortality: grade 1, 0%; grade 2, 1.3%; grade 3, 5.3%) than the previously reported model based on the presence/absence of portal hypertension.

**Conclusions:**

This new grading system is a simple method for prediction of the postoperative long-term and short-term outcomes.

## Introduction

Most of the patients with hepatocellular carcinoma (HCC) have underlying liver disease [1]. Therefore, precise preoperative evaluation of the patient’s liver function is essential to avoid postoperative liver failure and mortality. Many attempts have been made to develop criteria of liver function for safe liver resection [2–5]. Portal hypertension (PH) is listed as a contraindication for liver resection in Western countries, and the indocyanine green (ICG) test is widely used in East Asian countries to decide whether patients with HCC are candidates for surgery [6–13].

As a result of recent advances in surgical techniques and perioperative management, liver resection has become safer, and aggressive surgical resection for HCC has been proposed [14–18]. Therefore, the surgical strategy for HCC patients needs to be selected based on more sensitive and accurate criteria than the Child-Pugh score, model for end-stage liver disease (MELD) score, liver function analysis based on ICG R15 alone, or the presence/absence of PH [4,5,6,13].

Recently, Johnson *et al*. [19] proposed a new evidenced-based liver function grading system based on the serum albumin and bilirubin levels, the so-called ALBI grading system. However, liver resection for HCC is normally limited to patients with normal bilirubin levels [1,12]. Therefore, the usefulness of the ALBI grade in surgical candidates may be simply based on the serum albumin level.

We hypothesized that the combination of serum albumin and ICG R15 may allow simple, but precise evaluation of the liver function and more accurate prediction of the short- and long-term outcomes in surgical candidates. In this study, we used the data from multi-institutional international databases of patients undergoing liver resection to identify the factors that independently influence the survival and postoperative outcomes in patients with resectable HCC. The grades determined based on a combination of albumin and ICG R15, named the Albumin-Indocyanine Green Evaluation (ALICE) grading system, were clearly correlated with the survival and postoperative mortality and morbidity in patients with HCC undergoing liver resection.

## Patients and Methods

Elective liver resections for HCC carried out at major centers for the management of HCC, including two from Japan, one from the United Kingdom, and one from Switzerland were included in the analysis. The demographic, clinical and pathological data related to this study were extracted and analyzed retrospectively from an anonymized, prospectively collected database at each institution. No additional data were collected from the participants for this study other than that included in the original database. Although this study protocol was not reviewed by an ethics committee at each of the participating institutions, the collection and registration of the original database was performed according to the regulations and with the approval of ethics committee at each institution (The University of Tokyo, Saitama Cancer Center, University Hospital Centre Hospitalier Universitaire Vaudois, and University College London). The extent of hepatectomy required was evaluated according to the extent of disease progression, the liver function status, and the general condition of the patient. The status of disease progression and resectability were assessed by imaging studies such as contrast-enhanced computed tomography, magnetic resonance imaging, and ultrasonography. Liver function impairment associated with the underlying liver disease was assessed by liver biochemistry tests, Child-Pugh classification, and measurement of ICG R15 [12]. The ICG R15 value was measured within 3 months before surgery. In patients who underwent portal vein embolization, the ICG R15 value after portal vein embolization was used for the analysis. At the European centers, portal venous pressure was also evaluated preoperatively.

Macroscopic vascular invasion was defined as tumor thrombosis in the second-order branch and/or the first order branch of the portal vein, or major hepatic vein, according to the Japanese staging system [20]. Survival was measured from the date of surgery to the date of death or date of last follow-up. In the Japanese centers, PH was defined according to the criteria proposed by the Barcelona group for patients in whom the hepatic venous pressure gradient was not measured (ie, the presence of esophageal varices and/or a platelet count of <100,000/μL in association with splenomegaly) [21]. The presence/absence of PH was available in 1488 Japanese Child-Pugh A patients. Postoperative complications were recorded and graded according to the Dindo-Clavien classification [22]. Ascitic complication was defined as higher than grade II postoperative ascites. Postoperative mortality was defined as any death during the postoperative hospital stay or within 90 days of surgery. The causes of death were liver failure in nine patients, pulmonary complications in three patients, acute renal failure in two patients, sudden cardiac arrest in one patient, and aggressive HCC recurrence in two patients. Major liver resection was defined as liver resection involving more than 3 Couinaud’s segments.

### Statistical analysis

Categorical variables were analyzed using the X2 test or Fisher’s exact test, as appropriate. Continuous variables were analyzed using the Wilcoxon rank-sum test. The entire Japanese cohort (n = 1868) was randomly divided into two groups, the training set (n = 932) and the validation set (n = 936) stratified by the institution. To identify the prognostic factors for the future model, a multivariable analysis was performed of the data from the training set using a Cox proportional hazards model and the backward elimination procedure. A p-value less than 0.10 was set as the cutoff value for the elimination. The following 13 variables were examined as potential risk factors: age (y/o), sex, Child-Pugh class, serum albumin (g/L), log_10_bilirubin (μmol/L), log_10_ICG R15(%), presence/absence of macroscopic vascular invasion, serum alpha-fetoprotein level (ng/mL), tumor number, tumor size (cm), presence/absence of R1/2 resection, presence/absence of satellite nodules, and the grade of differentiation of the cancer cells. Since the Log_10_(ICG R15) showed a normal distribution for the entire cohort, this parameter was used instead of the ICG R15.

Cox regression analysis was performed on the data from the Japanese training set to establish an equation. By splitting the linear predictor at the 25th and 90th percentiles, three survival groups were generated. Using this classification, patients with HCC were classified as low-, medium- or high-risk patients, corresponding to the linear predictor value in the lowest 25th percentile, 25th and 90th percentile, and the highest 10th percentile, respectively. The overall survival curves were determined using the Kaplan-Meier method and compared using the log-rank test. The discriminatory performances of the ALICE grading system, Child-Pugh grading system and the model based on the presence/absence of PH were calculated and compared using Harrell’s C and Somers’ D statistics. All statistical analyses were 2-tailed and p-values less than 0.05 were considered to indicate statistical significance. The SAS software, version 9.3 (SAS Institute Inc., Cary, NC), was used for developing the ALICE score, randomization, receiver operating characteristic (ROC) curve analysis, and calculating the Harrell’s C and Somers’s D scores. For other analyses, the JMP 11 software (SAS Institute Inc., Cary, NC) was used.

## Results

The patient characteristics of the two Japanese institutions and the European institutions are shown in Table 1. In the European institutions, the presence of macroscopic vascular invasion, PH, and Child-Pugh B were considered as contraindications for surgical resection.

**Table 1:** Characteristics of the cohorts.

Patient characteristics	Japan (Center 1)	Japan (Center 2)	Europe (Multicenter)
Total number of patients	1466	402	70
Accrual period	1994–2011	1989–2014	2008–2014
Age (years)*	66 (59–72)	68 (63–73)	63 (55–70)
Gender (Male/female)**	1169/297 (80/20)	316/86 (79/21)	60/10 (86/14)
Child-Pugh class (A/B)	1265/201 (86/14)	373/29 (93/7)	70/0 (100/0)
Presence of macroscopic vascular invasion	110 (7.5)	31 (7.7)	0 (0)
Serum albumin (g/L)	37 (34–40)	39 (36–42)	43 (39–45)
ICG retention rate at 15 min (%)	14.2 (9.3–20.7)	13.2 (9.5–19.4)	9.9 (5.7–15.1)

* Median (IQR),

**Number (%) IQR, interquartile range;

ICG, indocyanine green.

Multivariable Cox regression analysis carried out on the data from the Japanese training set to determine the risk factors for the overall survival identified serum albumin (g/L) (hazard ratio [HR] 0.95, 95% confidence interval [CI] 0.92–0.97, P < 0.001), log_10_ICG R15 (%) (HR 2.76, 95% CI 1.75–4.37, P < 0.001), presence/absence of macroscopic vascular invasion (HR 2.50, 95% CI 1.79–3.44, P < 0.001), presence/absence of satellite nodules (HR 1.89, 95% CI 1.45–2.44, P < 0.001), and presence/absence of R 1/2 resection (HR 2.69, 95% CI 1.86–3.79, P < 0.001) as significant risk factors (Table 2). Of note, the log_10_bilirubin (μmol/L) was not identified as a significant variable.

**Table 2:** Multivariable Cox regression analysis of the data of the Japanese training cohort to identify prognostic factors associated with the survival.

Risk factors	P value	Hazard ratio (95% confidence interval)
Serum albumin (g/L)	< 0.001	0.95 (0.92–0.97)
Log_10_ ICG R15 (%)	< 0.001	2.76 (1.75–4.37)
Macroscopic vascular invasion	< 0.001	2.50 (1.79–3.44)
Satellite nodules	< 0.001	1.89 (1.45–2.44)
R1/2 resection	< 0.001	2.69 (1.86–3.79)

ICG R15, indocyanine green retention rate at 15 minutes.

We confined our model to the serum albumin and log_10_ICG R15(%) to focus on the variables related to the liver function. A Cox regression equation based on only the serum albumin and log_10_ICG R15(%) was built for the Japanese training set, and the equation for the linear predictor was as follows: linear predictor = (0.663 × log_10_ICG R15 [%])—(0.0718 × albumin [g/L]). Then the patients were classified into three prognostic groups, that is, ALICE grades 1 to 3 based on the values of the linear predictor, as follows; linear predictor value ≤ -2.20, ALICE grade 1; linear predictor value -2.20 to ≤ -1.39, ALICE grade 2; linear predictor value more than -1.39, ALICE grade 3.

**Fig 1:**
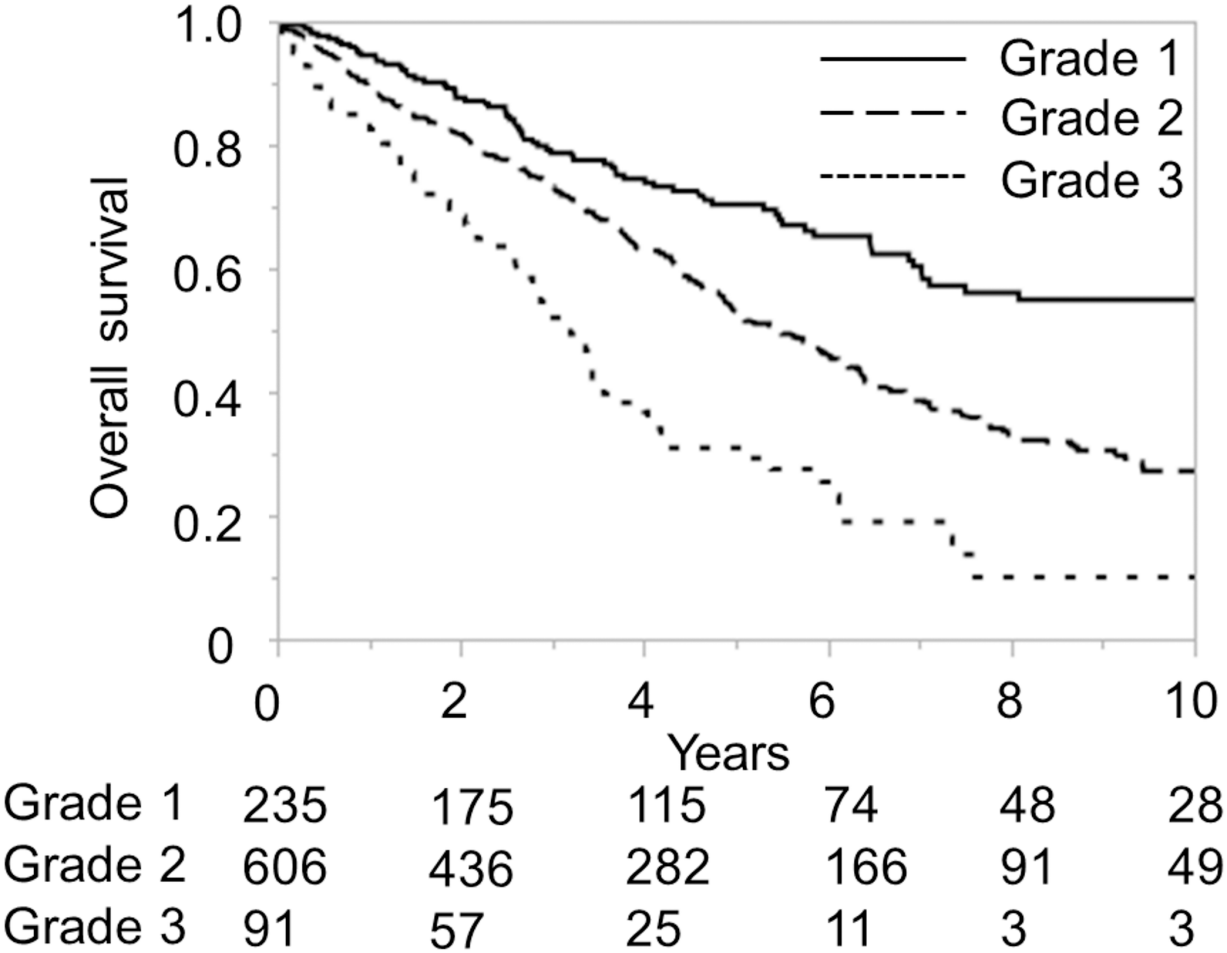
Overall survival according to the Albumin-Indocyanine Green Evaluation (ALICE) grade in the Japanese training cohort. Numbers below the x-axis indicate the number of patients at risk.

**Fig 2:**
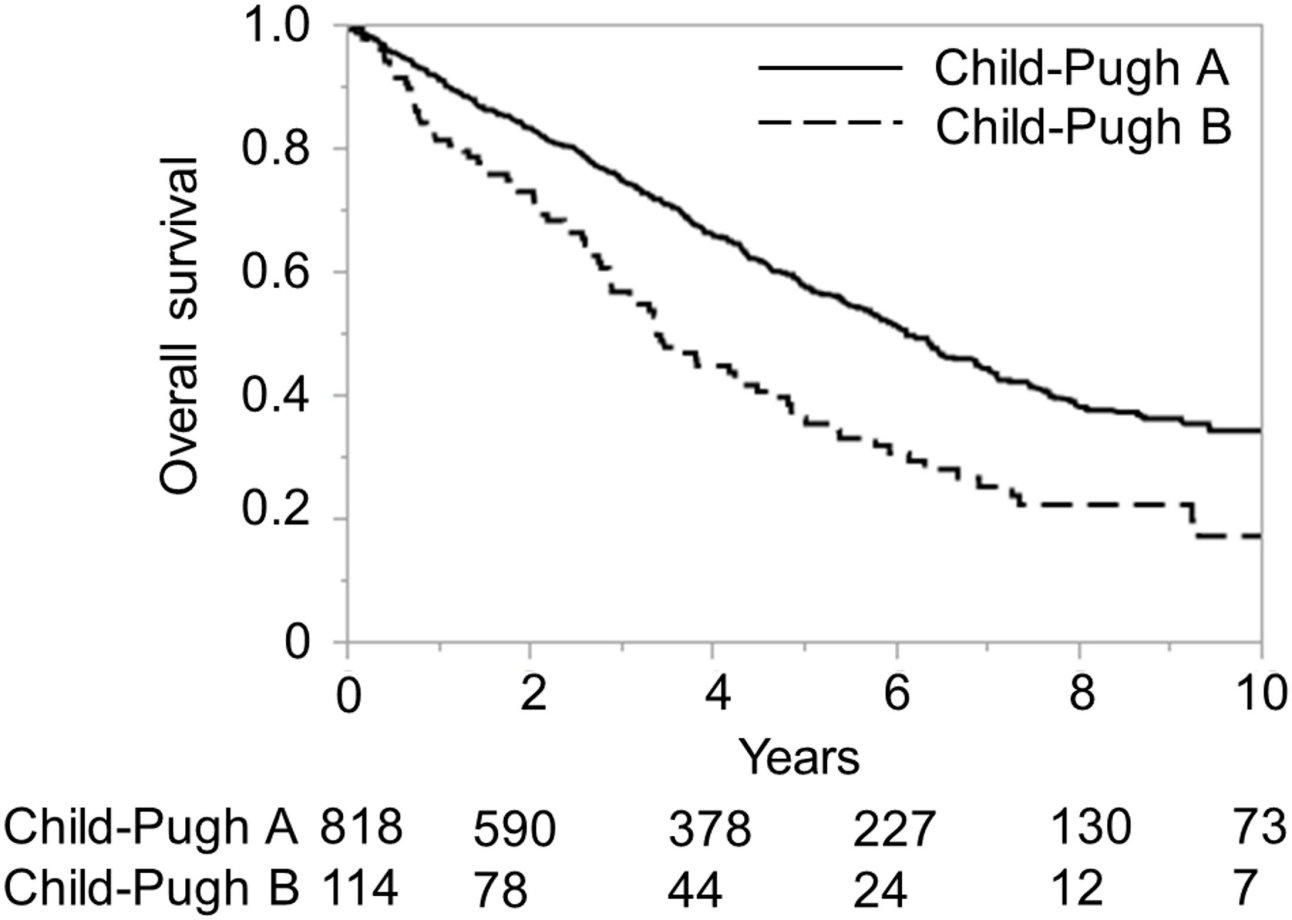
Overall survival according to the Child-Pugh grade in the Japanese training cohort. Numbers below the x-axis indicate the number of patients at risk.

**Fig 3:**
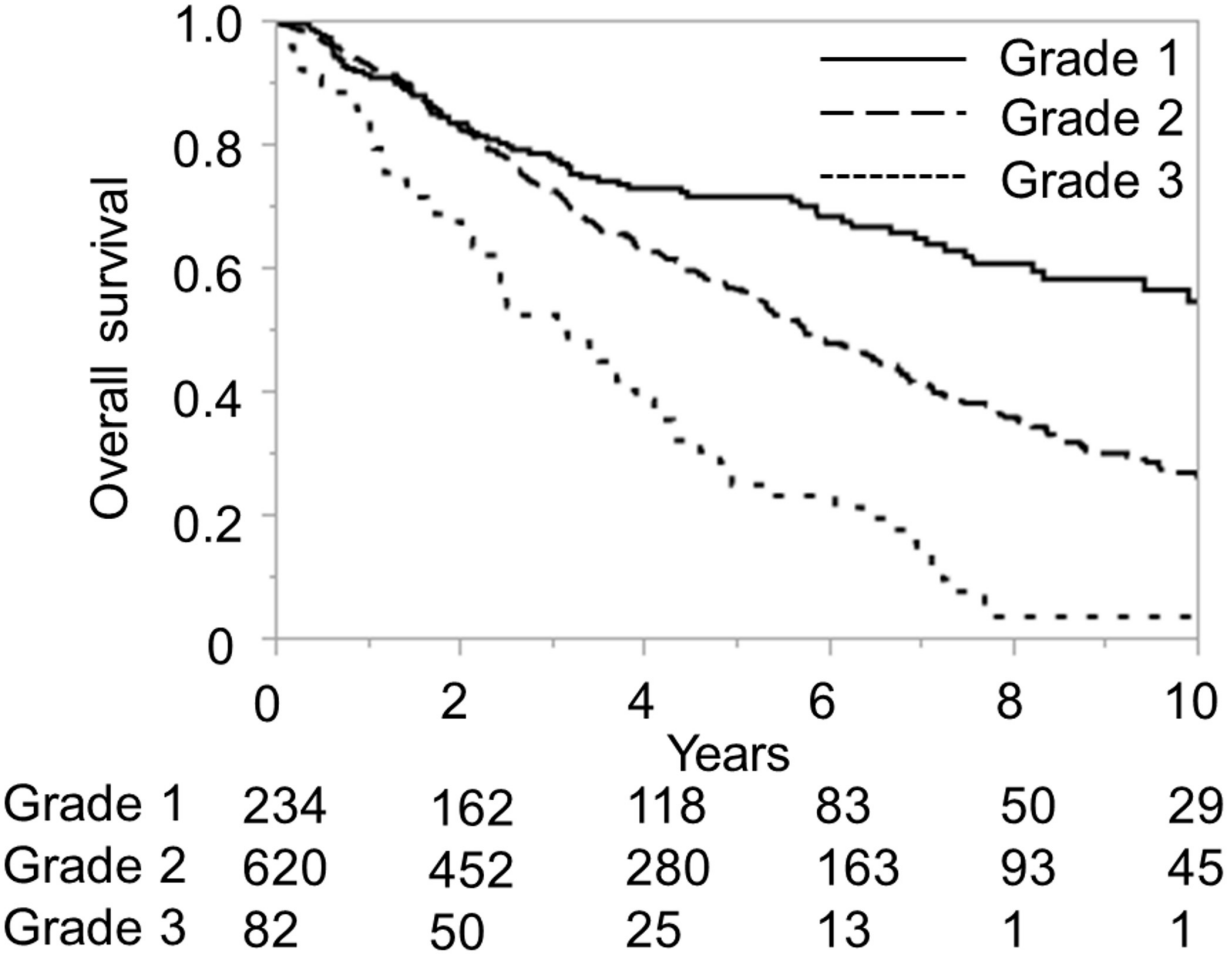
Overall survival according to the Albumin-Indocyanine Green Evaluation (ALICE) grade in the Japanese validation cohort. Numbers below the x-axis indicate the number of patients at risk.

**Fig 4:**
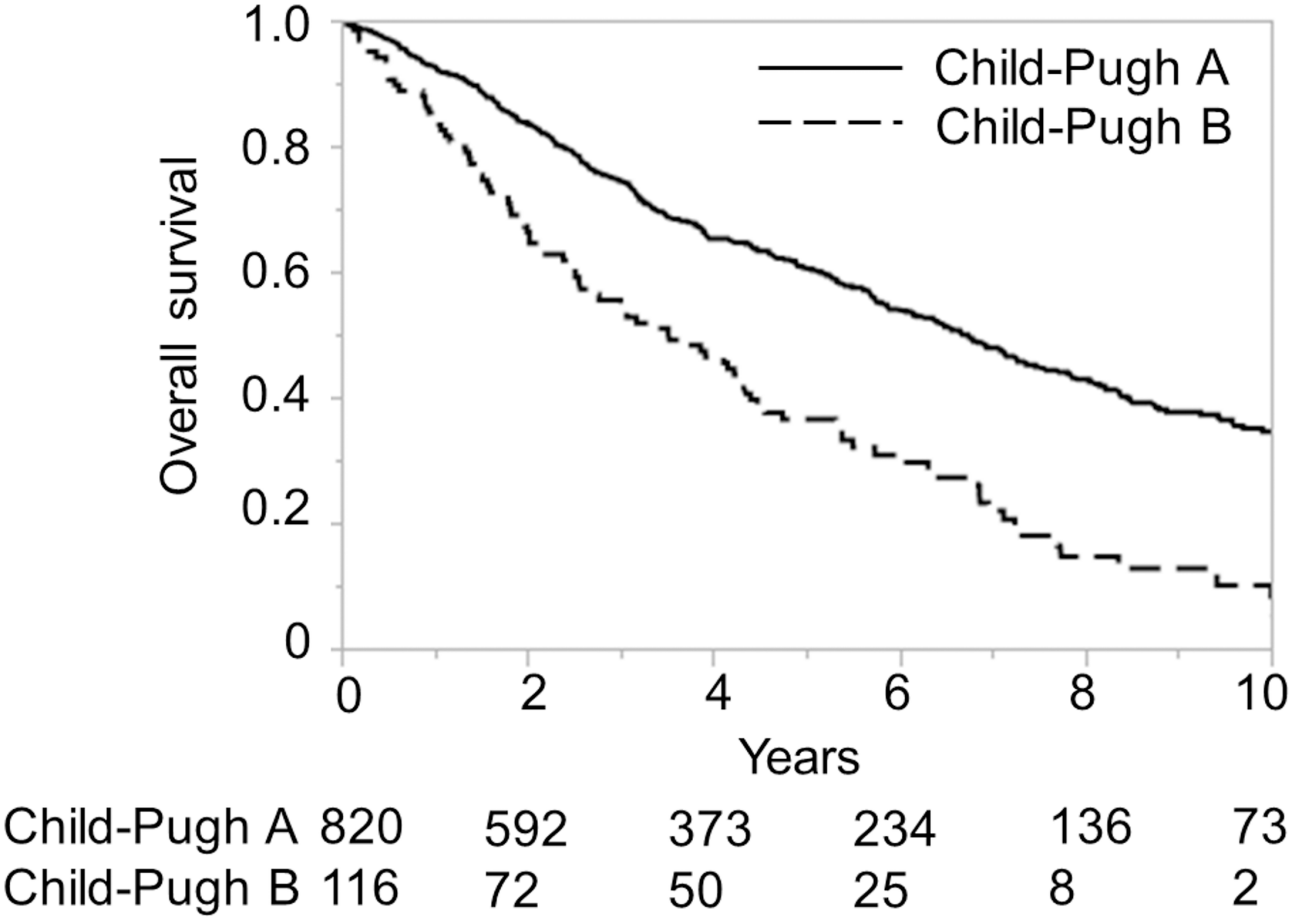
Overall survival according to the Child-Pugh grade in the Japanese validation cohort. Numbers below the x-axis indicate the number of patients at risk.

**Fig 5:**
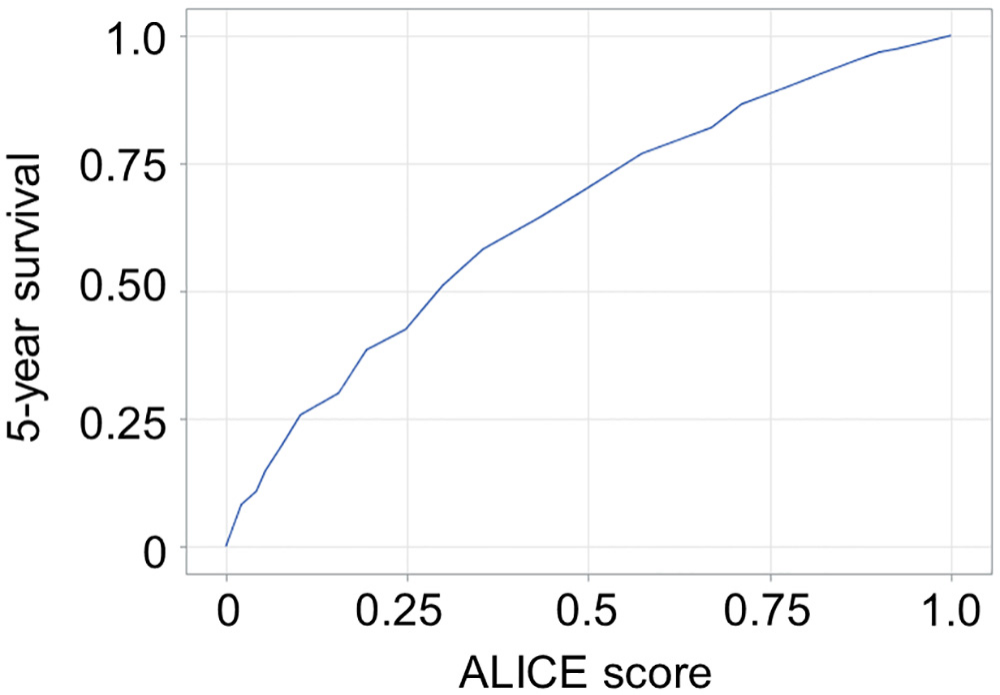
ROC curve of Albumin-Indocyanine Green Evaluation (ALICE) score in the Japanese training cohort.

The ALICE grading system was then applied to the training and validation sets of the Japanese cohort and compared with the Child-Pugh grading system for the same datasets. The Kaplan-Meier curve showed equally good discrimination between the three ALICE prognostic groups and the two Child-Pugh grade (A/B) groups (Figs 1–4). An ROC curve analysis of the ALICE score revealed that a universally good prediction for the whole population in the training cohort (Fig 5). The Harrell’s C and Somers’ D scores were similar, being 0.66 and 0.32 vs. 0.65 and 0.30, in the training set, 0.64 and 0.29 vs. 0.68 and 0.35 in the validation set, respectively. The median survival times (MSTs) of the ALICE grade 1, grade 2 and grade 3 patients were 11.8 y, 5.55 y and 3.27 y in the training set, and 11.9 y, 5.71 y and 3.08 y in the validation set, respectively. When the Child-Pugh grading system was applied, the MSTs in the Child-Pugh class A and B patients were 6.18 y and 3.39 y in the training set, and 6.75 y and 3.48 y in the validation set, respectively.

**Fig 6:**
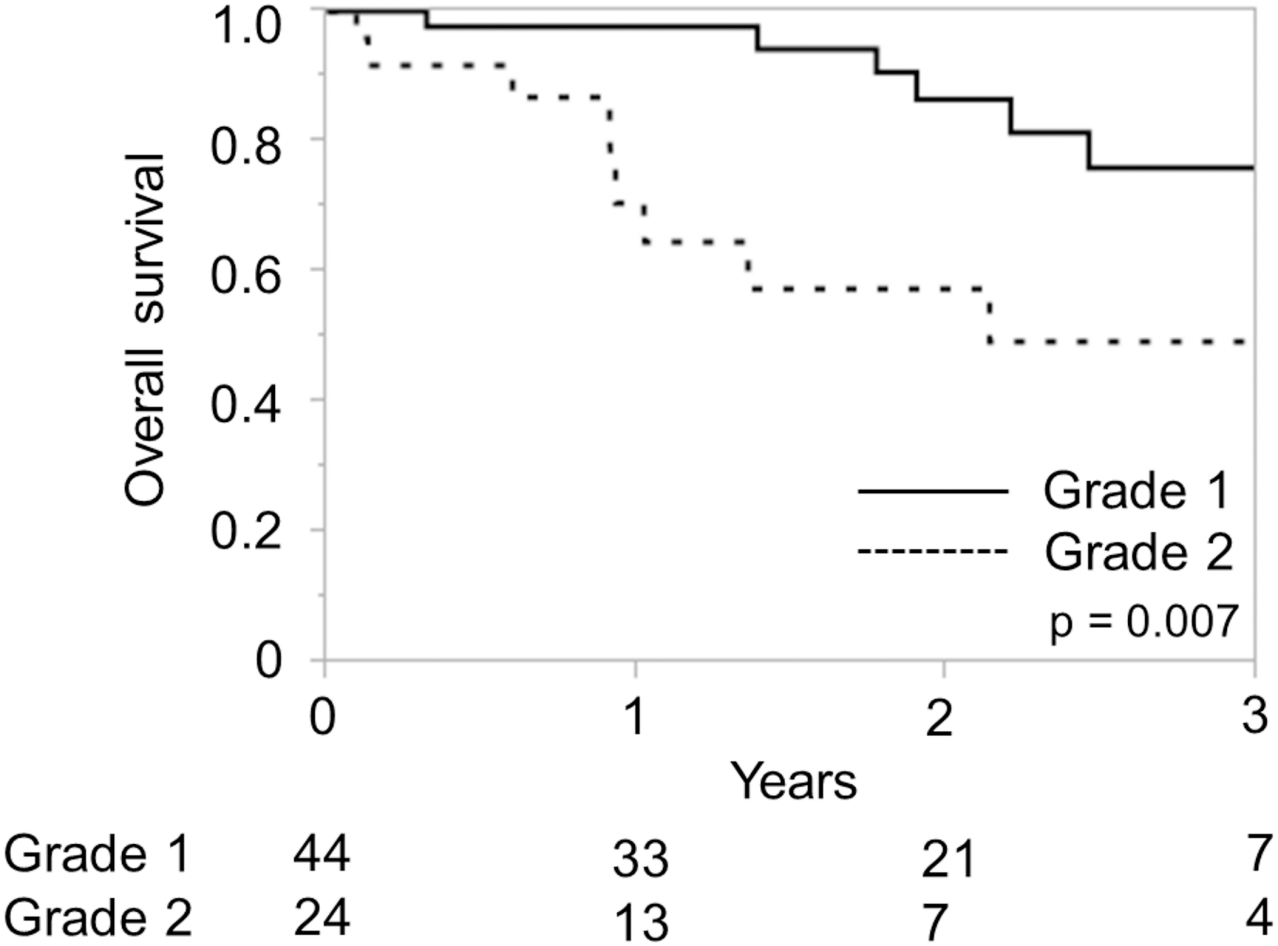
Overall survival according to the Albumin-Indocyanine Green Evaluation (ALICE) grade in the European cohort. Numbers below the x-axis indicate the number of patients at risk.

The new ALICE grading system was then applied to the European cohort. Since only 2 patients were classified as ALICE grade 3, we compared the overall survival between ALICE grade 1 and grade 2 patients; the overall survival was significantly longer in the grade 1 group as compared with that in the grade 2 group (P = 0.007), confirming the validity of the new grading system in western populations (Fig 6).

**Fig 7:**
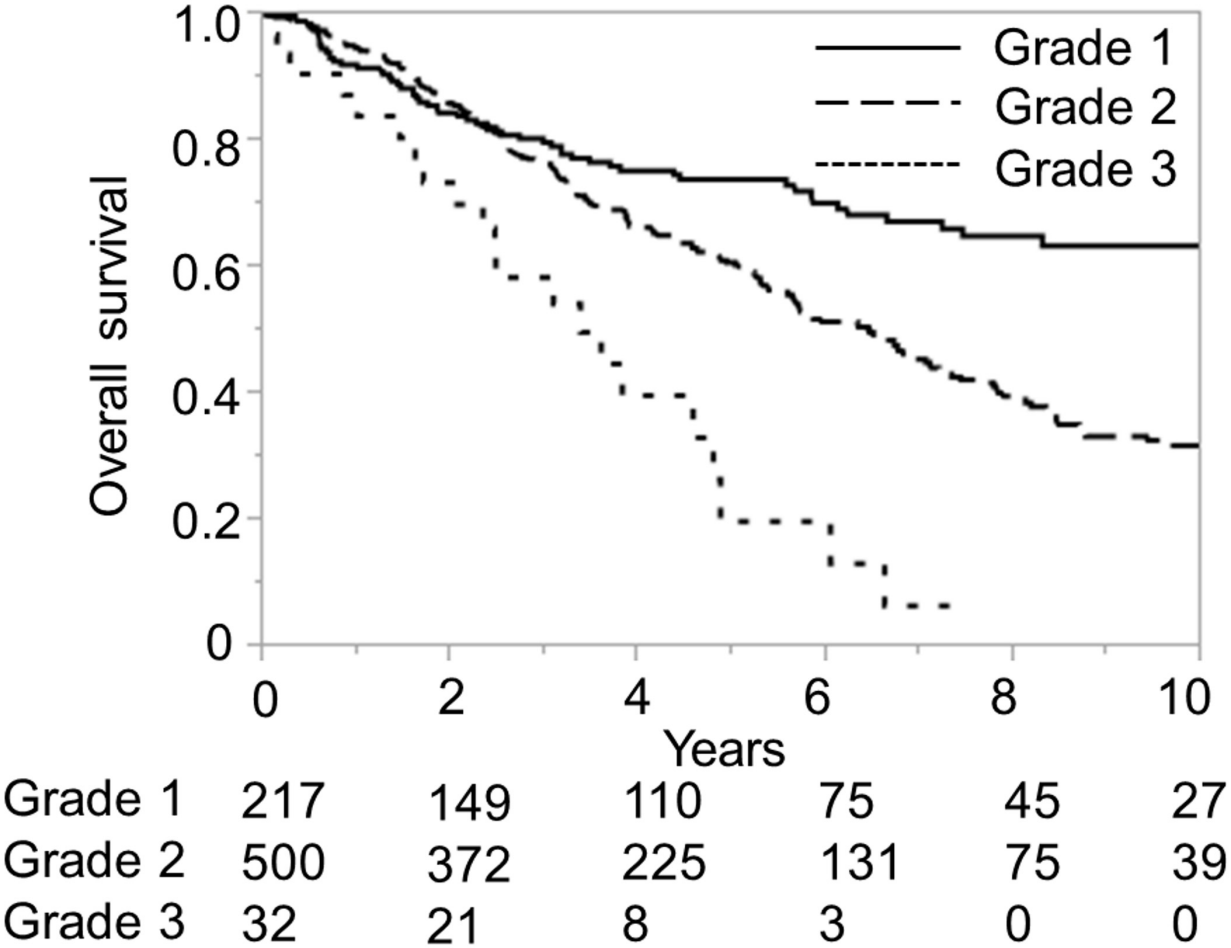
Overall survival according to the Albumin-Indocyanine Green Evaluation (ALICE) grade in a Japanese Child-Pugh A validation cohort. Numbers below the x-axis indicate the number of patients at risk.

**Fig 8:**
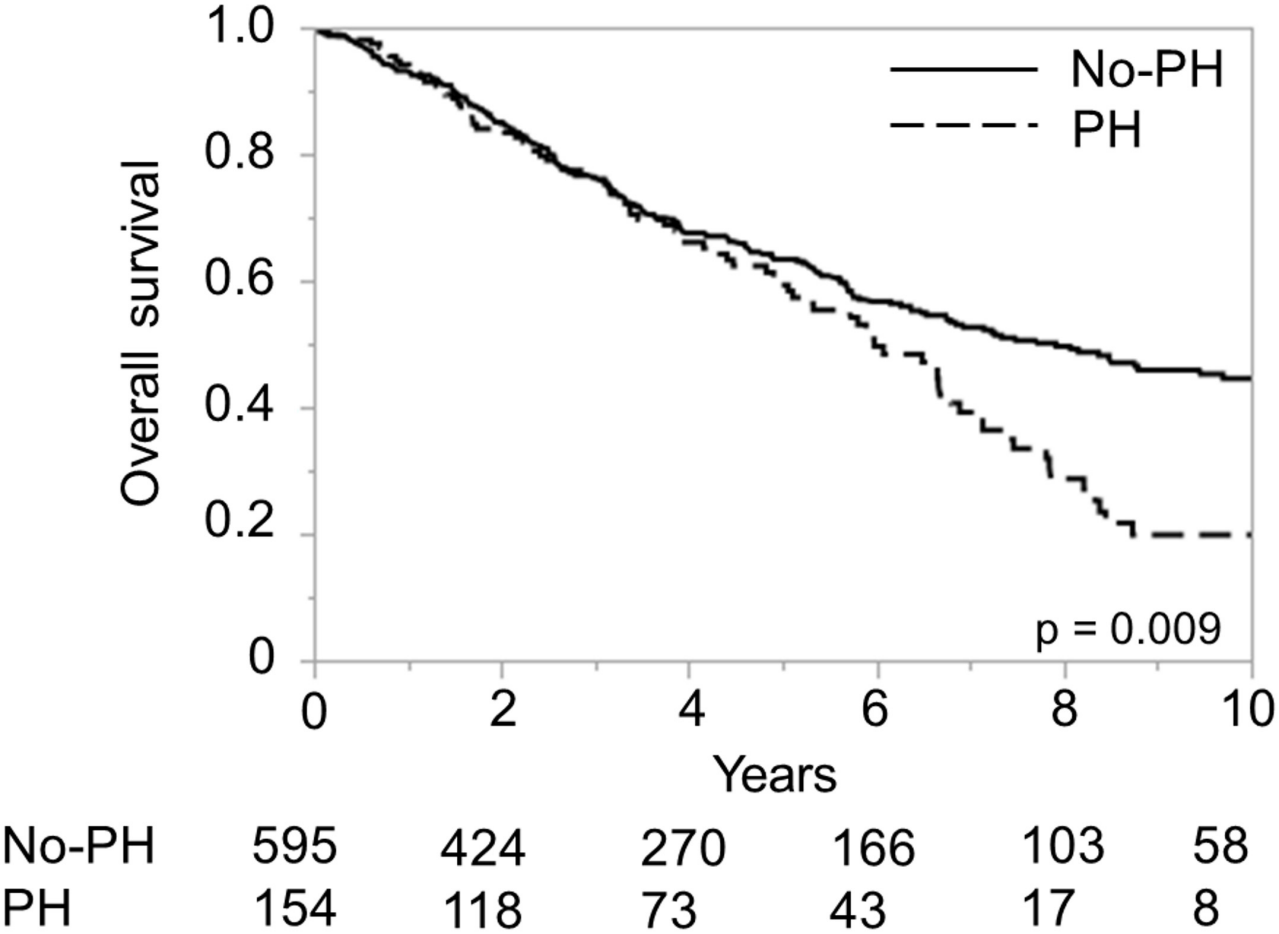
Overall survival based on the presence/absence of portal hypertension (PH) in a Japanese Child-Pugh A validation cohort. Numbers below the x-axis indicate the number of patients at risk.

As the presence of PH is considered to be a significant risk factor for a poor prognosis in Child-Pugh A patients [9], we compared the ALICE grading system to the model based on the presence/absence of PH in Child-Pugh A patients of the validation dataset. The Kaplan-Meier curve showed that the ALICE grading system allowed better discrimination of the risk than model based on the presence/absence of PH (Figs 7 and 8). The Harrell’s C and Somers’ D scores were better for the ALICE grading system than for the model based on the presence/absence of PH (0.61 and 0.23 vs. 0.54 and 0.08, respectively). The MSTs in the patients with ALICE grade 1, grade 2 and grade 3 were 12.5 y, 6.45 y, and 3.37 y, respectively, while those in the group with and without PH were 8.03 y and 6.03 y, respectively.

In order to analyze the predictive power of the ALICE grade for the short-term outcome, we analyzed the postoperative outcomes according to the ALICE grade and presence/absence of PH in the Child-Pugh A patients of the Japanese cohort (Table 3). Consistent with a previous report [9], the group with PH showed a significantly higher rate of ascites, and therefore a higher overall rate of complications and a longer hospital stay. The ALICE grading system also allowed the patients to be stratified according to the risk of ascites complication, overall rate of complications and length of hospital stay. In addition, the need for red blood cell transfusion and the mortality rate increased significantly as the ALICE grade became worse (transfusion requirement: grade 1, 5.0%; grade 2, 7.9%; grade 3, 14.7%; mortality rate: grade 1, 0%; grade 2, 1.3%; grade 3, 5.3%); such stratification was not possible in the model based on the presence/absence of PH (Table 3).

**Table 3:** Postoperative outcomes according to the Albumin-Indocyanine Green Evaluation (ALICE) grade and presence/absence of portal hypertension in the Child-Pugh A patients of the entire Japanese cohort.

	ALICE grade 1n = 437	ALICE grade 2n = 976	ALICE grade 3n = 75	P values (2 vs. 3)	non-PH groupn = 1174	PH groupn = 314	P values
Operative time (min)*	320 (245–414)	340 (265–450)	335 (260–510)	P = 0.509	340 (260–435)	340 (255–460)	P = 0.341
Blood loss (ml)	530 (280–900)	670 (380–1128)	810 (380–1490)	P = 0.076	600 (330–1052)	684 (428–1320)	P < 0.001
RBC transfusion required**	22 (5.0)	77 (7.9)	11 (14.7)	P = 0.041	88 (7.5)	22 (7.0)	P = 0.769
Median hospital stay (d)	14 (11–17)	16 (13–21)	20 (14–26)	P = 0.010	15 (12–19)	17 (14–24)	P < 0.001
Presence of complication	72 (16.5)	232 (23.8)	28 (37.3)	P = 0.009	248 (21.1)	84 (26.8)	P = 0.033
Ascites	9 (2.1)	63 (6.5)	12 (16.0)	P = 0.002	54 (4.6)	30 (9.6)	P < 0.001
90-day mortality	0 (0)	13 (1.3)	4 (5.3)	P = 0.008	15 (1.3)	2 (0.6)	P = 0.343

* Median (IQR),

**Number (%) IQR, interquartile range;

RBC, red blood cells; PH, portal hypertension.

**Fig 9:**
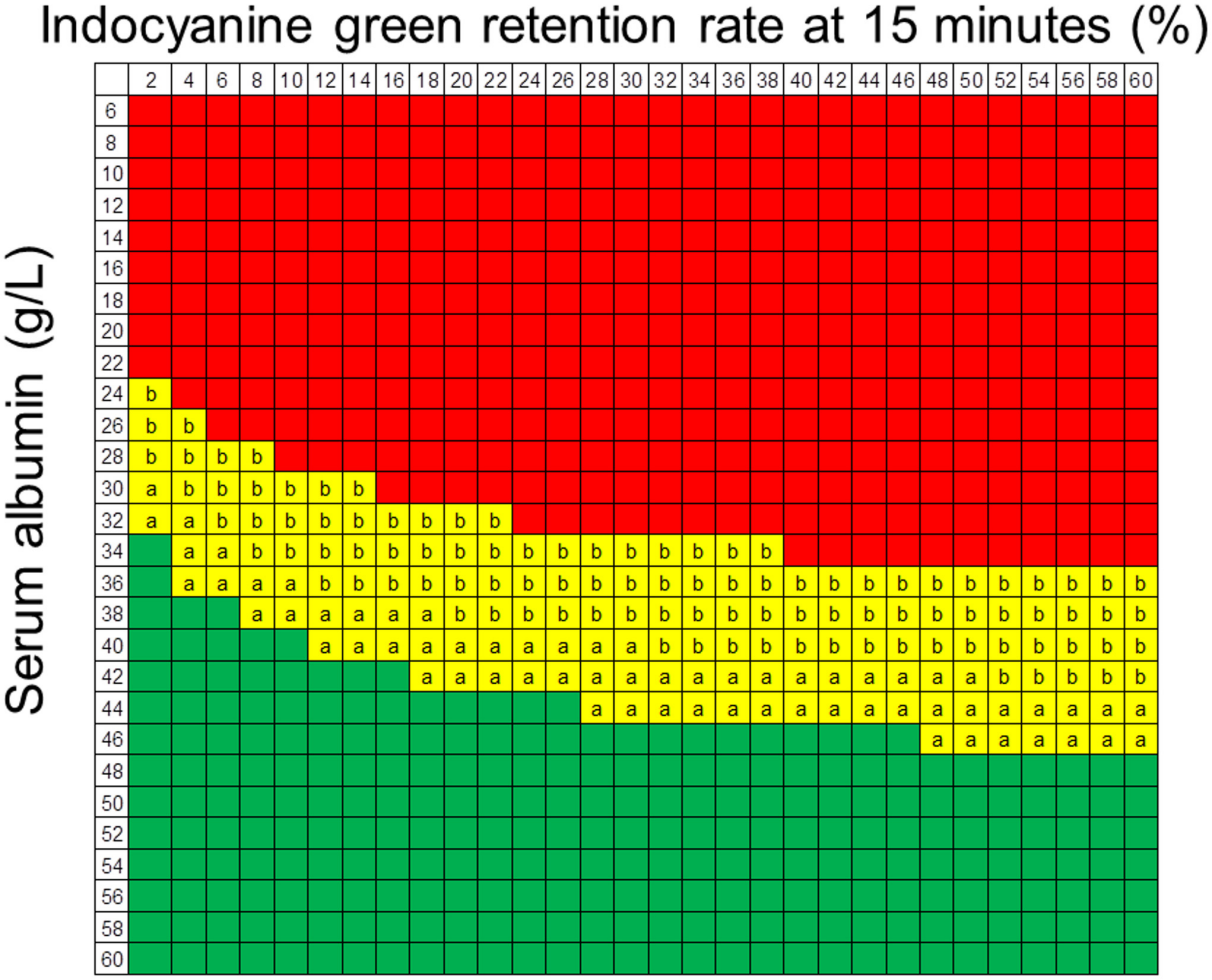
Heat map showing the Albumin-Indocyanine Green Evaluation (ALICE) grade. ALICE grade 1: green, ALICE grade 2a: yellow a, ALICE grade 2b: yellow b, ALICE grade 3: red.

**Fig 10:**
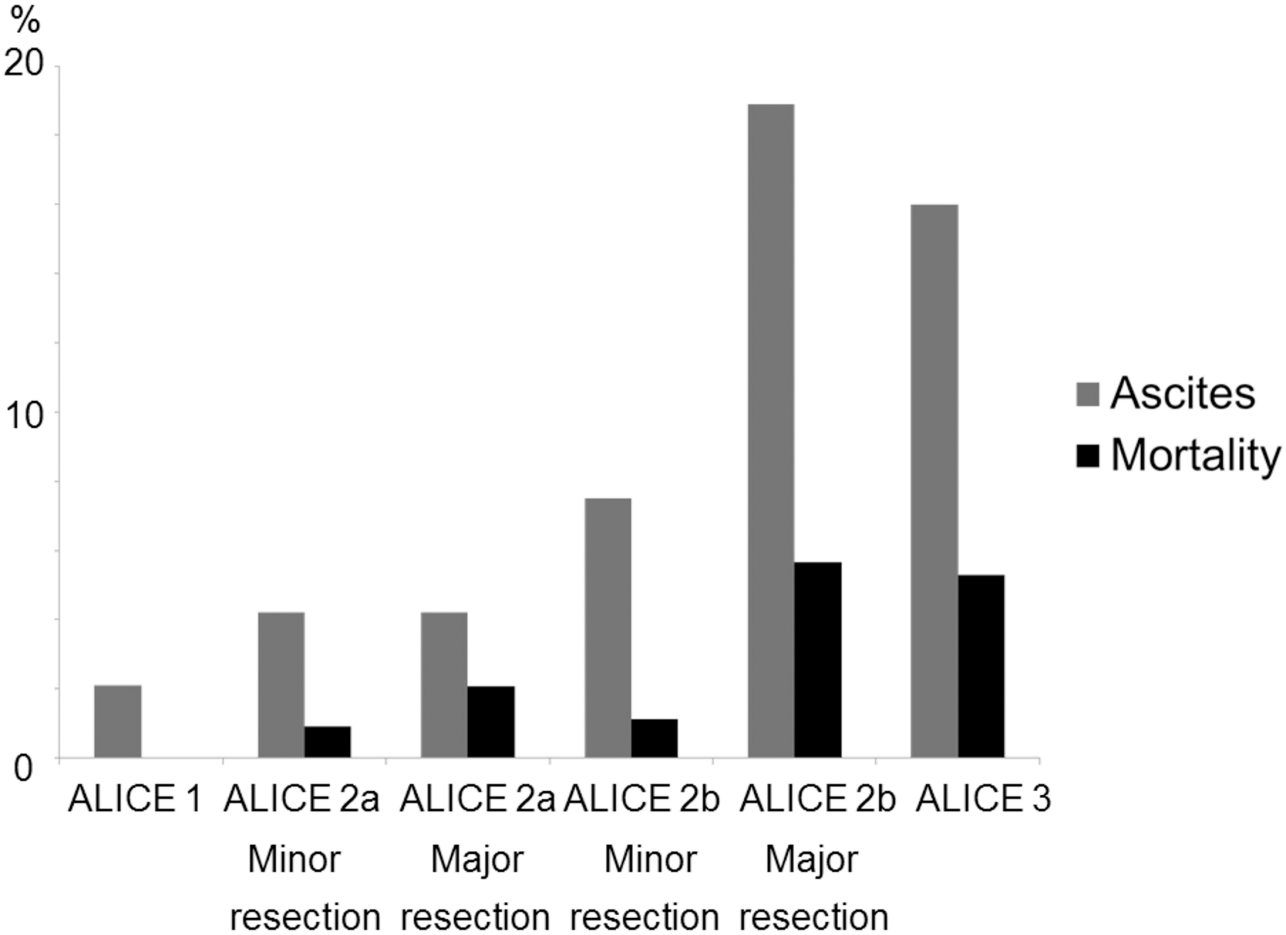
Incidence of ascites and mortality rate according to the Albumin-Indocyanine Green Evaluation (ALICE) grade.

A heat map using the 25th and 90th percentiles as the cut-off values for the ALICE grade revealed that most of the subjects with ALICE grade 1 had normal liver function, while those with ALICE grade 3 had poor liver function approximating Child-Pugh grade B (Fig 9). The extent of liver resection should be carefully considered in patients with ALICE grade 2. Therefore, we evaluated the extent of resection in ALICE grade 2 patients. We divided the patients with ALICE grade 2 into 2 groups using the median linear predictor (-1.88) as a cut-off value. Thus, the patients were classified into two groups (ALICE grades 2a and 2b) based on the values of the linear predictor as follows: ALICE grade 2a, linear predictor value of -2.20 to ≤ -1.88; ALICE grade 2b, linear predictor value of more than -1.88 to ≤ -1.39. The rates of PH in each grade were as follows: ALICE grade 1, 11.0% (48/437); ALICE grade 2a, 18.3% (88/481); ALICE grade 2b, 29.1% (144/495); and ALICE grade 3, 45.3% (34/75). Although major resection, i.e. more than 3 Couinaud’s segments, did not affect the incidence of ascites or the mortality rate in the ALICE grade 2a group (ascites: minor resection,18/433 [4.2%]; major resection, 2/48 [4.2%]; mortality rate: minor resection, 4/433 [0.9%]; major resection, 1/48 [2.1%]), major resection in the ALICE grade 2b group had a significantly higher incidence of ascites and mortality rate (ascites: minor resection, 33/442 [7.5%]; major resection, 10/53 [18.9%]; mortality rate: minor resection, 5/442 [1.1%]; major resection, 3/53 [5.7%]), similar to the results for patients with ALICE grade 3 (Fig 10).

## Discussion

The current study revealed that the new simple evidence-based model incorporating the serum albumin level and ICGR15 could be at least as useful for predicting the long-term outcome in HCC patients undergoing liver resection as the Child-Pugh grade. This new grading system was also useful for further stratification of Child-Pugh A patients. In addition, the ALICE grade was a better predictor of the postoperative long-term and short-term outcomes than the risk class based on the presence/absence of PH. Major liver resection among ALICE grade 2b patients was correlated with significantly higher morbidity and mortality rates.

**Fig 11:**
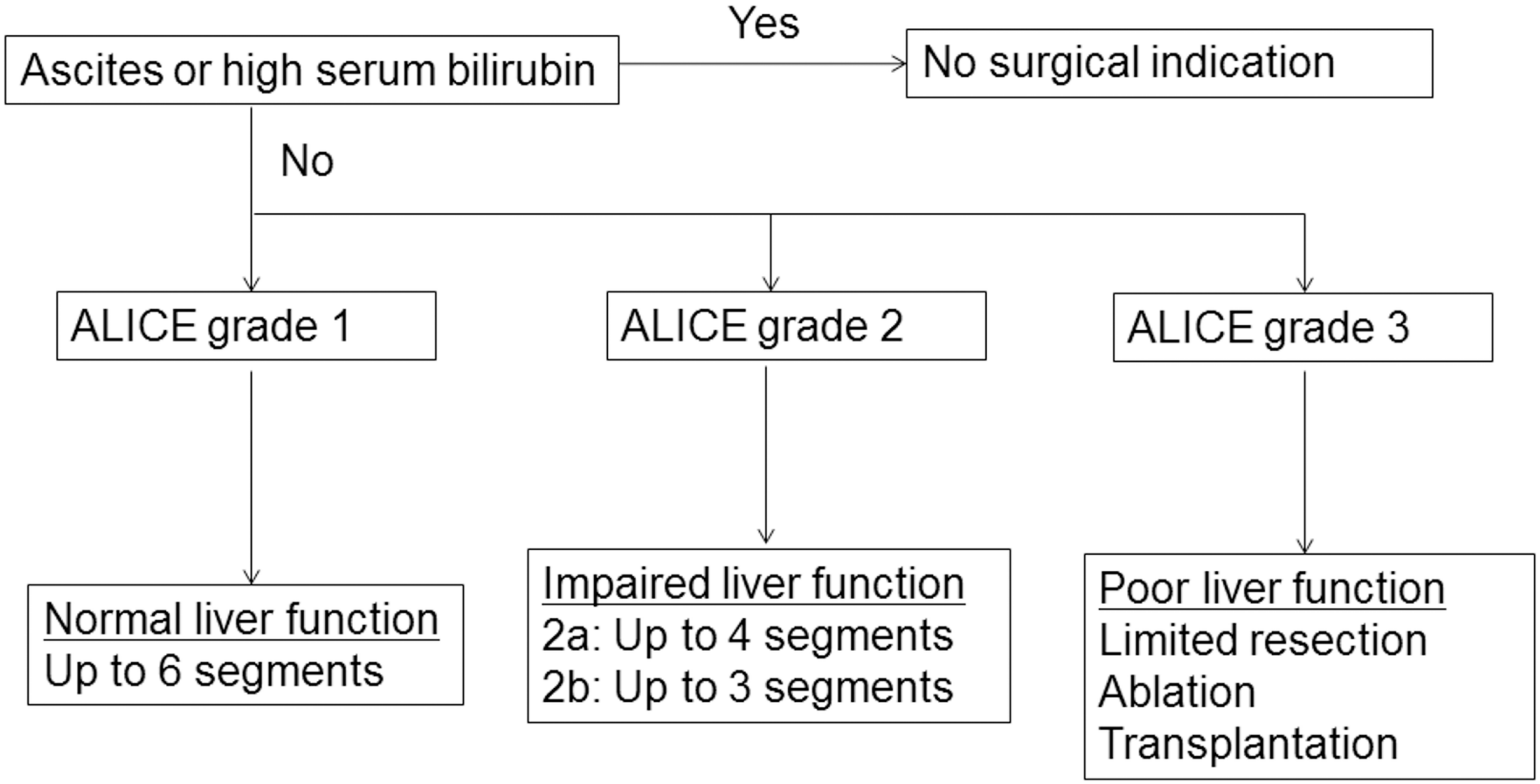
Proposed surgical treatment strategy for hepatocellular carcinoma patients according to the Albumin-Indocyanine Green Evaluation (ALICE) grade.

The proposed surgical strategies for HCC patients according to the ALICE grade are shown in Fig 11. Since ALICE grade 3 was associated with a poor prognosis and a high risk of postoperative liver failure, surgical resection in subjects classified as ALICE 3 should be considered carefully. The extent of liver resection should be limited in ALICE grade 3 patients, and ablation therapy or liver transplantation could be suitable alternatives where the tumor status is appropriate. In the patients with ALICE grade 1, since the risk of postoperative liver failure is extremely low and the MST is more than 10 years, surgical resection rather than liver transplantation is recommended. Patients in this group can be considered as having normal liver function and aggressive surgery such as trisectionectomy can be attempted. Since liver function is impaired in patients with ALICE grade 2, the extent of liver resection should be limited to 4 Couinaud’s segments, such as a right hepatectomy or an extended left hepatectomy. Since major resection was associated with high morbidity and mortality rates in ALICE grade 2b patients, liver resection should preferably be limited to 3 Couinaud’s segments, such as a left hepatectomy or sectionectomy.

As compared to the ALBI grade, in our data, the serum bilirubin level showed no significant association with the postoperative survival. This could be attributable to the fact that liver resection was confined to patients with normal serum bilirubin levels. However, Johnson et al. [19] demonstrated the effectiveness of the ALBI grade in predicting the postoperative survival in HCC patients undergoing liver resection. This is probably due to the effect of the serum albumin level, and indeed, the ALBI grade was also effective in our study population (data not shown). These results indicate that the serum albumin level is a strong predictor of the liver function.

Evaluation for the presence or absence of PH has been proposed in western countries, while measurement of the ICG R15 has been proposed in eastern countries [1]. This study clearly demonstrated that ALICE grading is superior to classification based on the presence/absence of PH for stratification of the prognosis and prediction of postoperative liver failure. The algorithm based on the ALICE grade modified the criteria proposed by Makuuchi et al. [12,13] by adding albumin, which is considered to be the most important variable influencing the Child-Pugh grade or the ALBI grade in HCC patients undergoing surgical resection. Therefore the ALICE grade can be considered as a combination of the Makuuchi criteria and the Child-Pugh grading system.

One of the limitations of this study was its retrospective nature. Since the extent of liver resection was based on the ICG R15 value and the Makuuchi criteria in the Japanese cohort, this may have caused a significant bias in our results. Although we confirmed the usefulness of ALICE grading in western populations, the population size of the western cohort was relatively small, because ICG R15 is not routinely measured in most western countries. Furthermore, differences in ethnicity may be one of the reasons that PH only had small impact on postoperative survival and the rate of complications. Therefore, these findings need to be confirmed in a prospective study carried out in a large number of western subjects.

In conclusion, this new grading system based on the serum albumin and ICG R15 is a simple and objective system that is useful for predicting the postoperative long-term and short-term outcomes after surgical resection for HCC. This new system could be potentially used worldwide for surgical decision making in patients with HCC.
